# Local and timely antimicrobial resistance data for local and national actions: the early implementation of an automated tool for data analysis at local hospital level in Thailand

**DOI:** 10.1093/jacamr/dlad088

**Published:** 2023-07-15

**Authors:** Voranadda Srisuphan, Preeyarach Klaytong, Chalida Rangsiwutisak, Kritiya Tuamsuwan, Phairam Boonyarit, Direk Limmathurotsakul

**Affiliations:** Health Administration Division, The Office of Permanent Secretary, Ministry of Public Health, Nonthaburi 11000, Thailand; Mahidol-Oxford Tropical Medicine Research Unit, Faculty of Tropical Medicine, Mahidol University, Bangkok 10400, Thailand; Mahidol-Oxford Tropical Medicine Research Unit, Faculty of Tropical Medicine, Mahidol University, Bangkok 10400, Thailand; Health Administration Division, The Office of Permanent Secretary, Ministry of Public Health, Nonthaburi 11000, Thailand; Health Administration Division, The Office of Permanent Secretary, Ministry of Public Health, Nonthaburi 11000, Thailand; Mahidol-Oxford Tropical Medicine Research Unit, Faculty of Tropical Medicine, Mahidol University, Bangkok 10400, Thailand; Nuffield Department of Medicine, Centre for Tropical Medicine and Global Health, University of Oxford, Oxford OX3 7LG, UK; Department of Tropical Hygiene, Faculty of Tropical Medicine, Mahidol University, Bangkok 10400, Thailand

## Abstract

**Background:**

In low- and middle-income countries (LMICs), hospitals can rarely utilize their own antimicrobial resistance (AMR) data in a timely manner.

**Objectives:**

To evaluate the utility of local AMR data generated by an automated tool in the real-world setting.

**Methods:**

From 16 December 2022 to 10 January 2023, on behalf of the Health Administration Division, Ministry of Public Health (MoPH) Thailand, we trained 26 public tertiary-care and secondary-care hospitals to utilize the AutoMated tool for Antimicrobial resistance Surveillance System (AMASS) with their own microbiology and hospital admission data files via two online meetings, one face-to-face meeting and online support. All meetings were recorded on video, and feedback was analysed.

**Results:**

Twenty-five hospitals successfully generated and shared the AMR reports with the MoPH by 28 February 2023. In 2022, the median frequency of hospital-origin bloodstream infections (BSIs) caused by carbapenem-resistant *Escherichia coli* (CREC) was 129 (range 0–1204), by carbapenem-resistant *Klebsiella pneumoniae* (CRKP) was 1306 (range 0–5432) and by carbapenem-resistant *Acinetobacter baumannii* (CRAB) was 4472 (range 1460–11 968) per 100 000 patients tested for hospital-origin BSI. The median number of all-cause in-hospital deaths with hospital-origin AMR BSI caused by CREC was 1 (range 0–18), by CRKP was 10 (range 0–77) and by CRAB was 56 (range 7–148). Participating hospitals found that the data obtained could be used to support their antimicrobial stewardship and infection prevention control programmes.

**Conclusions:**

Local and timely AMR data are crucial for local and national actions. MoPH Thailand is inviting all 127 public tertiary-care and secondary-care hospitals to utilize the AMASS. Using any appropriate analytical software or tools, all hospitals in LMICs that have electronic data records should analyse and utilize their data for immediate actions.

## Introduction

In high-income countries (HICs), timely use of local antimicrobial-resistance (AMR) data has been proven useful to monitor the impact of local and national actions to tackle AMR.^1–3^ In England, the local data on AMR, antimicrobial use, and infection prevention and control (IPC) are available through a publicly accessible web tool.^1^ This tool allows the local hospitals and policymakers to compare the ‘AMR local indicators’ profile between areas and over time.^1^ Many national authorities in HICs regularly publish AMR reports, including total number of cases and deaths, and frequency of infection (e.g. per 100 000 population and per 100 000 patient-days) by areas and over time.^2,3^

In low- and middle-income countries (LMICs), there is some national coordination for an AMR surveillance system; however, having a system that is able to generate local and reliable information on AMR and feedback to local hospitals in a timely manner is still a challenge. For example, in Thailand, 92 surveillance sites participated in the National Antimicrobial Resistance Surveillance Thailand (NARST) and 10 surveillance sites provided data to Global Antimicrobial Resistance Surveillance System (GLASS) in 2020.^4^ The NARST also makes a cumulative antibiogram for each organism from each participating hospital available online.^5^ However, the process takes time. In March 2023, only the cumulative antibiogram from each participating hospital up to 2020 is available online.^5^ The cumulative antibiograms are not stratified by infection origin, do not include other parameters (e.g. total number of cases and deaths, and frequency of infection) and could not be easily compared between hospitals or areas.

The AutoMated tool for Antimicrobial resistance Surveillance System (AMASS), an offline application to generate standardized AMR surveillance reports from routinely available microbiology and hospital data files, was developed and independently tested in seven hospitals in seven countries.^6^ The automatically generated reports stratify by infection origin into community origin and hospital origin based on the recommendations of WHO’s GLASS,^7^ and provided additional reporting on mortality (%) and total number of deaths with AMR and non-AMR bloodstream infections (BSIs). We recently reported the difference of frequency of AMR BSI between tertiary-care hospitals (TCHs) and secondary-care hospitals (SCHs) using the AMASS v2.0 and retrospective data from 2012 to 2015.^8^

The national action plan on AMR in Thailand aims to reduce AMR morbidity by 50%, and the analysis of baseline data from target hospitals is included in the plan.^9^ In addition, the data of patients with AMR infection can be used as measures/indicators to monitor and guide the antimicrobial stewardship (AMS)^10^ and IPC programmes.^11^ Therefore, we consider that the availability and timeliness of local AMR data is one of the most important aspects for local hospitals, national authorities and policymakers. Here, we aim to evaluate the utility of local AMR data generated by the AMASS in the real-world setting in Thailand.

## Methods

### Study setting

In 2022, Thailand had a population of 66.1 million, consisted of 77 provinces, and covered 513 120 km^2^. Health regions in Thailand were divided into 12 groups of provinces plus Bangkok, totalling 13 regions, using the concept of decentralization.^12^ Health Administration Division, Ministry of Public Health (MoPH) Thailand, supervised 127 public TCHs and SCHs in health regions 1 to 12 countrywide. These included 35 advanced-level referral hospitals (i.e. class A, with a bed count of about 500–1200 beds), 55 standard-level referral hospitals (i.e. class S, with a bed count of about 300 to 500) and 37 mid-level referral hospitals (i.e. class M1, with a bed count of about 180–300).^13^ Based on the WHO definitions, class A hospitals are generally TCHs and referred to as regional hospitals,^14^ and classes S and M1 are generally SCHs and referred to as provincial or large district hospitals. All class A and S hospitals, and most of class M1 hospitals, are equipped with a microbiology laboratory capable of performing bacterial culture using standard methodologies for bacterial identification and susceptibility testing provided by the Bureau of Laboratory Quality and Standards, Department of Medical Sciences, MoPH, Thailand.

### Early implementation of the AMASS in the real-world setting

From 16 December 2022 to 10 January 2023, on behalf of the Health Administration Division, MoPH, we invited and trained 26 TCHs and SCHs to utilize the AMASS with their own microbiology and hospital admission data files via two online meetings, one face-to-face meeting and online support. These 26 hospitals were invited because they were highly active in previous activities with the MoPH; 1 to 4 hospitals were selected from each health region, and the MoPH aimed to have them train other hospitals to utilize the AMASS in the next phase.

The first online meeting focused on (i) informing participants about the AMASS; (ii) teaching participants to be able to download, unzip and successfully run the AMASS with the example datasets provided in the AMASS;^6^ and (iii) informing participants about data files that they needed to export from their own hospital information system (HIS) and laboratory information system (LIS) to be used in the next session.

The second online meeting focused on supervising hospitals to run the AMASS with their own hospital datafiles; including how to (i) complete the data dictionary files of the AMASS; (ii) verify the log files to understand whether the AMASS automatically imported and analysed the data accurately or not; and (iii) read the AMR surveillance reports automatically generated by the AMASS. We emphasized that hospitals should not transfer raw patient-level data files to the study team.

The face-to-face meeting focused on (i) validation of the generated AMR reports; (ii) comparison of multiple AMR parameters (including proportion, frequency and total number of patients and deaths with hospital-origin BSI caused by carbapenem-resistant *Escherichia coli* (CREC), carbapenem-resistant *Klebsiella pneumoniae* (CRKP) and carbapenem-resistant *Acinetobacter baumannii* (CRAB) with other participating hospitals; (iii) and discussion on how to utilize the local and timely AMR reports generated by the AMASS for local actions.

For the online meetings, we requested the heads (or responsible persons) of each microbiology laboratory and HIS to participate. For the face-to-face meeting, we additionally requested representatives from AMS and IPC teams to participate.

In the second online and face-to-face meetings, we asked participants whether the data in their AMR reports generated by the AMASS were proportional to their workload (e.g. the total number of admissions, blood culture used and blood culture-positive specimens) and their AMR situation (e.g. proportion, frequency, and total number of patients and deaths with AMR BSI) they observed in their own hospitals. We also asked whether they found the AMASS and the AMR reports useful or not.

In the face-to-face meeting in January 2023, we asked each participating hospital to generate the AMR surveillance report using their own hospital admission and microbiology laboratory data files of the year 2022 and the AMASS v2.0.^15^

### Study design

This was a retrospective study describing the epidemiology of AMR among participating TCHs and SCHs in Thailand, and the utility of local and timely data generated by the AMASS. All meetings were recorded on video, and feedback was analysed by P.K. and K.T.

AMASS analysed the data and generated reports based on the recommendation of the WHO GLASS.^7^ Patient hospital number (HN) was automatically used as a record linkage between the microbiology and hospital admission data files. A deduplication process was automatically conducted in which only the first isolate of a species per patient per specimen type per survey period was included.

For AMR infections, we analysed the following organisms: MRSA, third-generation cephalosporin-resistant *E. coli* (3GCREC) and *K. pneumoniae* (3GCRKP), CREC, CRKP, carbapenem-resistant *Pseudomonas aeruginosa* (CRPA) and CRAB, which are in the WHO GLASS priority list of AMR bacteria and are locally important. Only blood culture isolates were included in the analysis of the AMASS v2.0 and in this study.

### Definitions

Community-origin (hospital-origin) BSIs were defined for patients in the hospital within (longer than) the first two calendar days of admission when the first blood specimen culture positive for a pathogen was taken, with calendar day one equal to the day of admission. Patients were considered at risk for hospital-origin BSI after they stayed in the hospital for more than two calendar days.

The proportion of AMR (%) was calculated as the percentage of patients with new AMR BSI over all patients with new BSIs for the pathogen of interest during the reporting period. The frequency of community-origin AMR BSI was calculated as the total number of new patients with community-origin AMR BSI per 100 000 patients tested for community-origin BSI (i.e. those who had the first blood culture collected within the first two calendar days of hospital admission)^6^ and per 100 000 admissions during the reporting period. The frequency of hospital-origin AMR BSI was calculated as the total number of new patients with hospital-origin AMR BSI per 100 000 patients tested for hospital-origin BSI (i.e. those who had the first blood culture collected after the first two calendar days of hospital admission),^6^ per 100 000 admissions and per 100 000 patient-days at risk for hospital-origin BSI during the reporting period. All-cause in-hospital mortality was defined using the discharge summary regularly completed by the attending physicians and reported to the MoPH.

### Statistical analysis

We compared all continuous variables using the Mann–Whitney test. *P* values were given to two significant figures, and no longer than four decimal places (e.g. *P* < 0.0001). Box and dot plots were used to illustrate the distribution of data. We used STATA (version 14.2; College Station, TX, USA) for the final statistical analyses and R version 4.0.5 for figures.

### Data availability

The anonymous AMR surveillance reports generated from participating hospitals are open access and available at https://doi.org/10.6084/m9.figshare.22339933.

### Ethics

Ethical permission for this study was obtained from the Institute for the Committee of the Faculty of Tropical Medicine, Mahidol University (TMEC 23-011). Individual consent was not sought from the patients as this was a retrospective study, and the Ethical and Scientific Review Committees approved the process.

## Results

### Baseline information of participating hospitals

**Figure 1.**
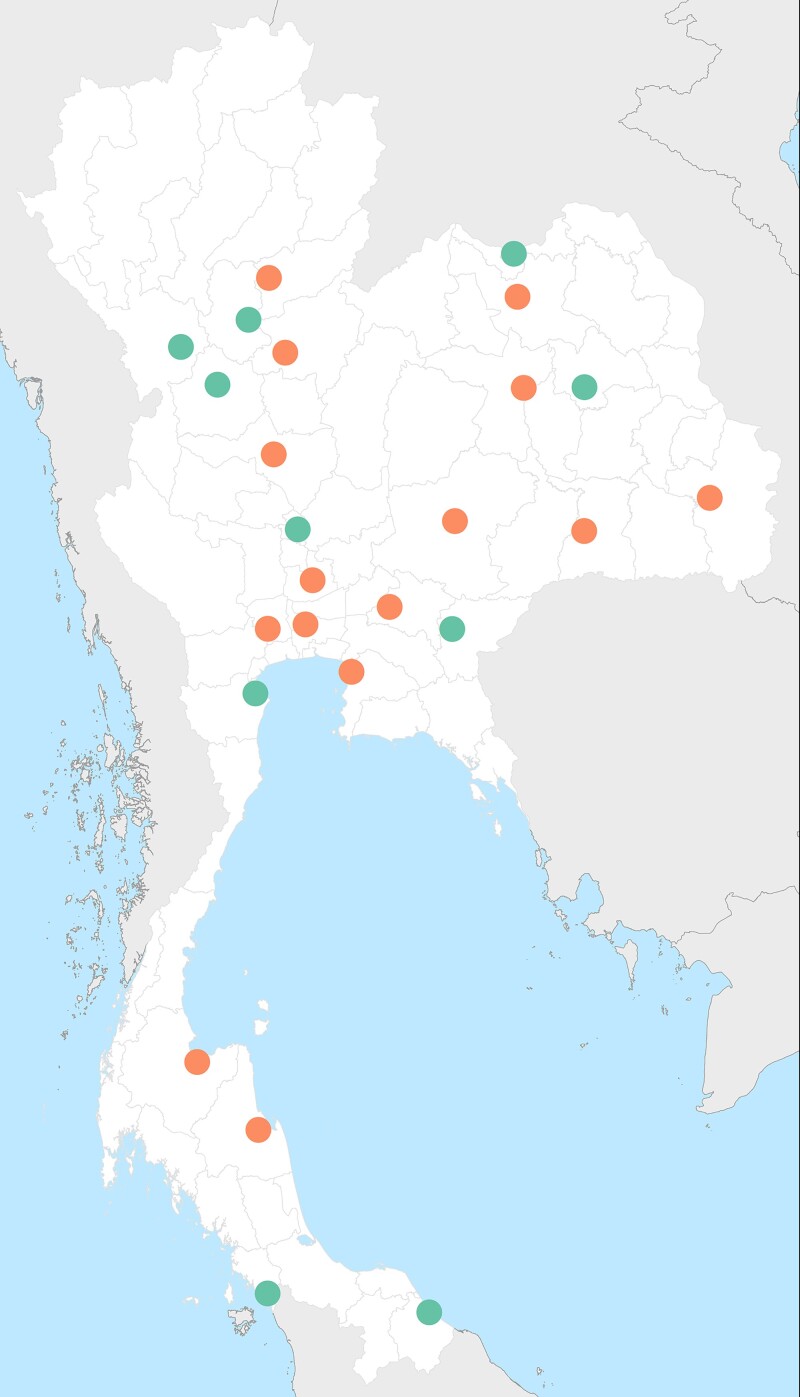
Location of 25 participating hospitals. Each dot represents a hospital. Orange dots represent TCHs and green dots represent SCHs.

Of 26 hospitals invited to the early implementation of the AMASS, all generated and shared their AMR surveillance reports for the year 2022 with the MoPH by 28 February 2023. However, the AMR report from anonymous hospital no. 1 was excluded because the data did not pass validation by the study team. The AMR reports of the other 25 hospitals were validated and included in further analysis (Figure 1).

These 25 hospitals were located in the health regions 2 to 12, with one to four hospitals per health region in Thailand. Of 25 hospitals, 15 (58%) and 10 (42%) were classified as TCHs and SCHs, respectively. Of 10 SCHs, 9 and 1 were classified as class S and M1 by the MoPH Thailand. The median bed number was 860 (range 527–1387) in TCHs and 414 (range 282–535) in SCHs (*P* = 0.0001; Table 1).

**Table 1.** Characteristics of 25 participating hospitals

Parameters in 2022^a^	SCHs (*n* = 10)	TCHs (*n* = 15)	*P* value
Bed number	414 (282–535)	860 (527–1387)	0.0001
Total number of hospital admissions	27 247 (16 380–46 384)	57 700 (32 647–103 085)	0.0001
Total patient-days	155 333 (95 665–263 300)	396 078 (201 087–692 547)	0.0001
Patient-days at risk for hospital-origin BSI (i.e. all patient-days >2 calendar days)^b^	101 444 (63 764–174 312)	282 167 (133 761–593 379)	0.0001
Total number of blood culture specimens	11 258 (5659–29 404)	35 633 (19 790–88 102)	0.0001
All-cause in-hospital mortality among all inpatients, %	3.9 (2.3–6.0)	5.2 (2.7–9.0)	0.040

a Data are presented as median (range).

b Patients were considered at risk for hospital-origin BSI after they stayed in the hospital for more than two calendar days.

### Proportion of AMR

In 2022, for community-origin BSI, the median proportion of MRSA was 6% (range 0%–23%), of 3GCREC was 34% (range 18%–49%), of 3GCRKP was 22% (range 10%–46%), of CREC was 1% (range 0%–25%), of CRKP was 7% (range 1%–20%), of CRPA was 14% (range 0%–79%) and of CRAB was 50% (range 13%–100%). The median proportion of AMR was not different between TCHs and SCHs for all pathogens (Figure S1, available as Supplementary data at *JAC-AMR* Online).

**Figure 2.**
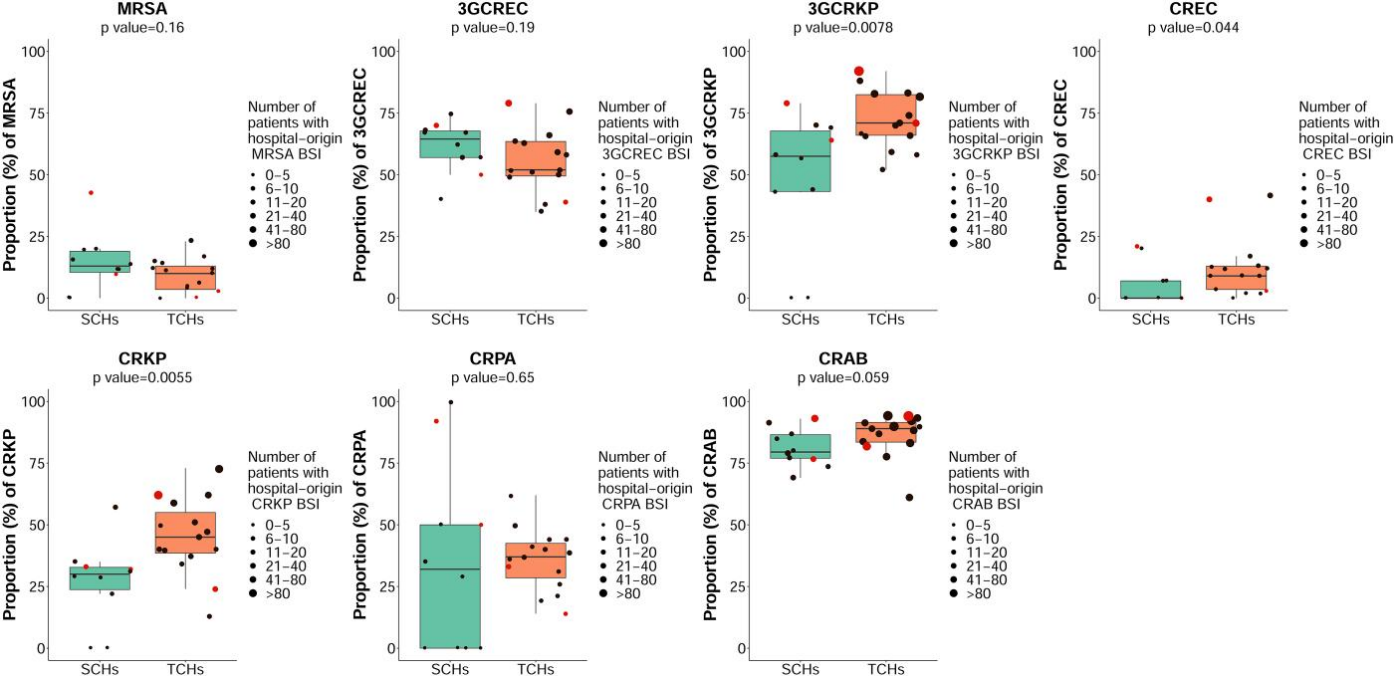
Proportion of AMR among patients with hospital-origin BSI in 15 TCHs and 10 SCHs in Thailand in 2022. Proportion of AMR (%) was calculated as the percentage of patients with new AMR BSI over all patients with new BSIs for the pathogen of interest during the reporting period. Each dot represents a hospital. Red dots represent anonymous hospitals no. 22 and 24 (among TCHs) and anonymous hospitals no. 16 and 25 (among SCHs).

For hospital-origin BSI, the median proportion of MRSA was 12% (range 0%–43%), of 3GCREC was 58% (range 35%–79%), of 3GCRKP was 67% (range 0%–92%), of CREC was 7% (range 0%–42%), of CREKP was 37% (range 0%–72%), of CRPA was 36% (range 0%–100%) and of CRAB was 87% (range 61%–94%). The median proportion of AMR for 3GCRKP, CREC and CRKP was higher in TCHs than in SCHs (Figure 2).

### Frequency of AMR BSI

In 2022, the median frequency of patients with community-origin BSI caused by MRSA per 100 000 patients tested for community-origin BSI was 60 (range 0–253), by 3GCREC was 1039 (range 333–2525), by 3GCRKP was 260 (range 79–527), by CREC was 50 (range 0–325), by CRKP was 75 (range 19–265), by CRPA was 41 (range 0–1556) and by CRAB was 137 (range 34–294). The median frequency of AMR BSI was not different between TCHs and SCHs for all pathogens (Figure S2).

**Figure 3.**
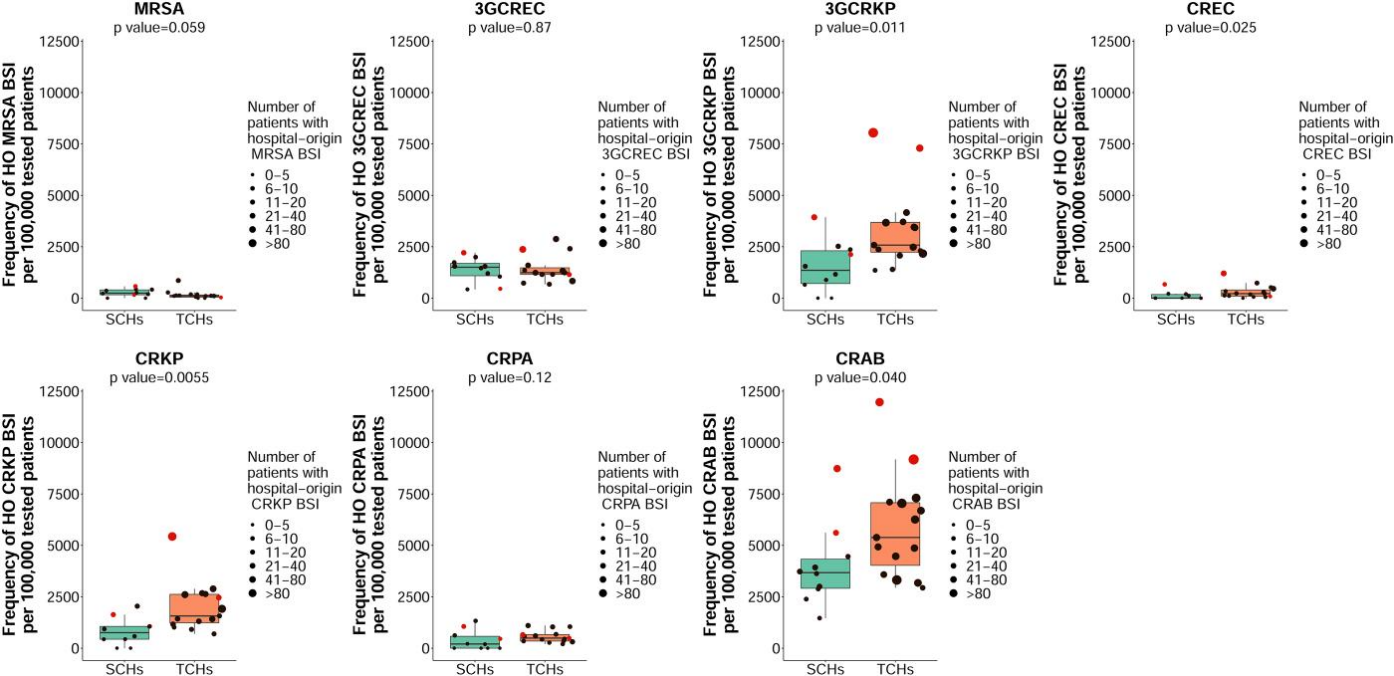
Frequency of hospital-origin AMR BSI per 100 000 patients tested for hospital-origin BSI in 15 TCHs and 10 SCHs in Thailand in 2022. Frequency of hospital-origin AMR BSI was calculated as the total number of new patients with hospital-origin AMR BSI per 100 000 patients tested for hospital-origin BSI (i.e. those who had the first blood culture collected after the first two calendar days of hospital admission). Each dot represents a hospital. Red dots represent anonymous hospitals no. 22 and 24 (among TCHs) and anonymous hospitals no. 16 and 25 (among SCHs).

The median frequency of hospital-origin BSI caused by MRSA per 100 000 patients tested for hospital-origin BSI was 132 (range 0–857), by 3GCREC was 1269 (range 427–2877), by 3GCRKP was 2363 (range 0–8045), by CREC was 129 (range 0–1204), by CRKP was 1306 (range 0–5432), by CRPA was 447 (range 0–1331) and by CRAB was 4472 (range 1460–11 968). The median frequency of AMR BSI for 3GCRKP, CREC, CRKP and CRAB was higher in TCHs than in SCHs (Figure 3).

As the frequency of hospital-origin CRAB BSI was highest among AMR pathogens under evaluation in our setting, we initially compared the frequency of hospital-origin CRAB BSI among participating hospitals. We noted that anonymous hospitals no. 22 and 24 (among TCHs) and anonymous hospitals no. 16 and 25 (among SCHs) had the highest frequency (Figure 3). We also observed that those four hospitals had a frequency of other hospital-origin AMR BSI, particularly CRKP, relatively higher compared to that of other hospitals.

We also estimated the frequency of hospital-origin AMR BSI per 100 000 admissions and per 100 000 patient-days at risk for hospital-origin BSI (data not shown), and similar findings were observed.

### All-cause in-hospital mortality (%) of patients with AMR BSI

The median all-cause in-hospital mortality (%) of patients with hospital-origin AMR BSI caused by MRSA was 42% (range 0%–100%), by 3GCREC was 33% (range 0%–67%), by 3GCRKP was 55% (range 24%–100%), by CREC was 50% (range 0%–100%), by CRKP was 57% (range 10%–100%) and by CRAB was 64% (range 26%–100%). Difference in the all-cause in-hospital mortality (%) of patients with AMR BSI caused by each AMR pathogen was not observed between TCHs and SCHs (all *P* > 0.10), except that the median all-cause in-hospital mortality of patients with community-origin CRKP BSI was higher in TCHs than in SCHs (39% versus 0%, *P* = 0.023).

### Total number of deaths with AMR BSI

**Figure 4.**
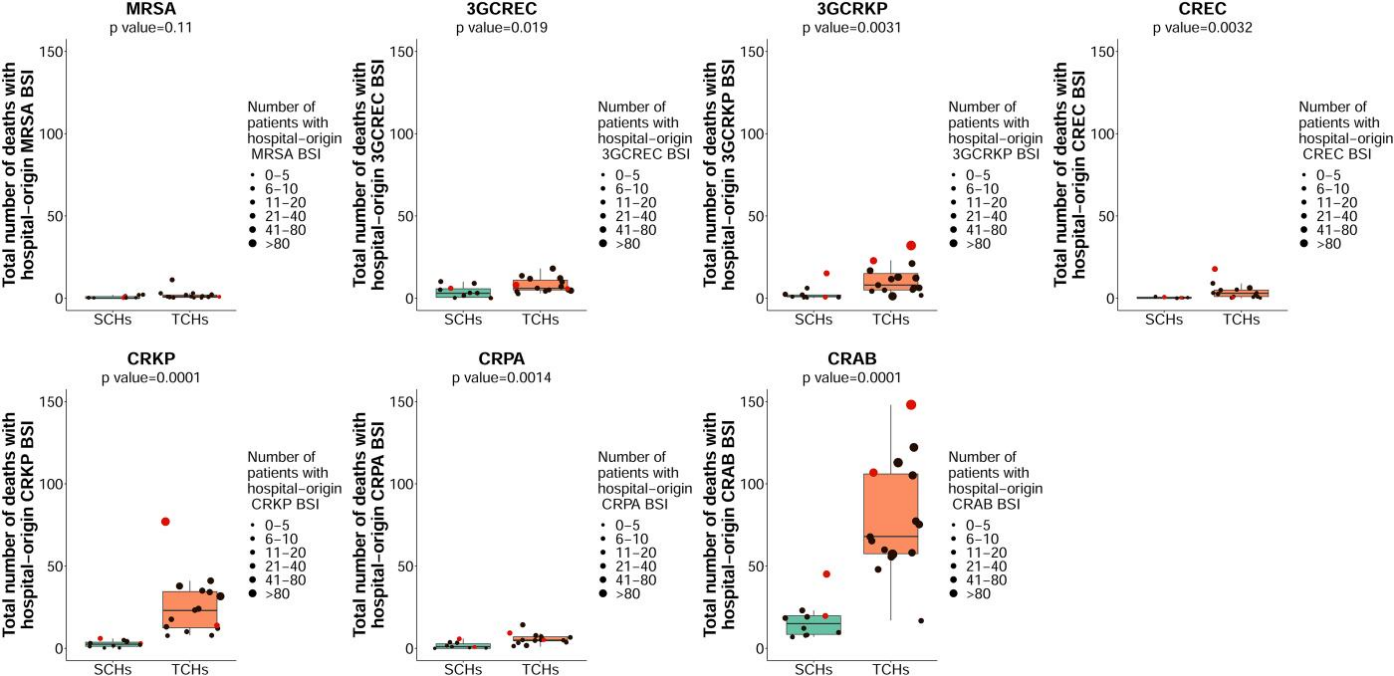
Total number of deaths with hospital-origin AMR BSI in 15 TCHs and 10 SCHs in Thailand in 2022. All-cause in-hospital mortality was used. Each dot represents a hospital. Red dots represent anonymous hospitals no. 22 and 24 (among TCHs) and anonymous hospitals no. 16 and 25 (among SCHs).

For the purpose of stimulating local actions, we compared total number of deaths with hospital-origin AMR BSI between hospitals (Figure 4). The median total number of deaths with hospital-origin MRSA was 1 (range 0–11), with 3GCREC was 5 (range 0–18), with 3GCRKP was 5 (range 0–32), with CREC was 1 (range 0–18), with CRKP was 10 (range 0–77), with CRPA was 4 (range 0–14) and with CRAB was 56 (range 7–148). We noted that anonymous hospitals no. 22 and 24 (among TCHs) and anonymous hospitals no. 16 and 25 (among SCHs) had high total number of deaths with hospital-origin AMR BSI for multiple organisms compared with other hospitals.

### Feedback on the data generated by the AMASS programme

A total of 26 microbiology laboratory staff, 14 infection control (IC) nurses, 7 pharmacists, 7 information technology staff, and 2 medical doctors participated in the training and provided feedback. All microbiology laboratory staff responded that the cumulative antibiograms obtained from the AMASS would be useful for their reports to their AMS and IPC teams and to national authorities. All medical doctors, IC nurses and pharmacists agreed that the summary data reported by the AMASS could be used to support their AMS and IPC programmes.

## Discussion

This study demonstrates the utility of local and timely AMR data generated by the AMASS for local and national actions in the real-world setting. The utility of the data is shown by the ability to compare epidemiology data of participating hospitals in a timely manner and by feedback from the participating hospitals. The MoPH Thailand is inviting all 127 public TCHs and SCHs to utilize the AMASS in 2023 and planning to update the AMR dashboard by using data generated from the AMASS nationwide.

The AMASS empowers hospitals to analyse, generate and share their own cumulative antibiograms, together with their total number of patients and deaths with AMR BSI, in a quick manner (i.e. within 2 months after the end of the year) and with minimal resources required (i.e. without the need for data experts with adequate skills in statistical software). The AMR data obtained highlight the significant burden of AMR infections in participating hospitals in 2022 and the urgency to step up actions against AMR infections countrywide.

At the local hospital level, the frequency of hospital-acquired AMR BSI and other data of patient outcomes (e.g. in-hospital mortality) can be used as measures/indicators to monitor and guide their AMS^10^ and IPC programmes.^11^ The observed numbers of patients and deaths with AMR BSI in their own hospitals last year can be used to communicate with all levels of healthcare workers, including the directors and executive boards of the hospitals, better than the modelling data, national estimates or global estimates.^16^ Some participating hospitals are generating their own AMR reports independently using the data from previous years (e.g. 2018, 2019, 2020 and 2021), discussing the findings among staff in their own hospitals, and sharing those reports with the MoPH.

At the national level, the ability to obtain and compare reliable AMR data between hospitals in a quick manner is crucial for designing interventions and resource allocation. If the AMASS were utilized by all TCHs and SCHs in Thailand, the policymaker could understand the situation of the AMR in the country, in each region and in each hospital much more clearly. Utilization of the AMASS can also support participating hospitals to join the WHO GLASS Thailand.

The future version of the AMASS aims to include automatic analysis of electronic drug prescription data and generate reports on diagnostic and antibiotic use practices. The availability of data of AMR burden, diagnostic practice and antibiotic use practice at the local hospital level nationwide will support the national action plan on AMR,^9^ the National Drug Policy and National Drug System Development Strategy,^17^ the Rational Drug Use Service Plan,^18^ and the national plan to achieve the sustainable development goals (SDGs) in Thailand.^19^

At the international level, the data from hospitals of all sizes in LMICs can be helpful for modelling studies estimating the global burden of AMR.^20^ The all-cause in-hospital mortality reported by the AMASS^6,21^ can be used by modelling studies together with studies using recommended methods for estimating attributable mortality of AMR BSI.^22–25^

The higher frequency of hospital-origin AMR BSI in TCHs compared with SCHs observed in this study is consistent with the previous study using the data of 49 hospitals from 2012 to 2015.^8^ The frequency observed in this study is relatively higher than that previously observed.^8^ The change of the AMR burden over time will be studied separately using the data of 127 public TCHs and SCHs in 2022.

The AMASS has some common issues that users face. Firstly, users need to change the Windows region settings from Thailand to either US or UK because the AMASS cannot run properly with the Windows region setting as Thailand. Secondly, dates must not be exported as date time variables. Users need to export dates as date variables or generate new variables in the Excel files as date variables prior to running the AMASS. Lastly, users need to complete the dictionary files using the log files as guidance. The completion of the dictionary files is crucial because it is not uncommon for each bacterial species to be recorded in the LIS with multiple names (e.g. *Escherichia coli*, *E. coli*, ESBL *E. coli* and ESBL-producing *E. coli*). For AMASS users, we emphasize the importance of completing the dictionary files and validating the AMR reports with log files, as well as comparing the data obtained in the report with their previously known workload (e.g. total number of hospital admissions and total number of blood culture specimens), their previous AMR reports (if available) and manual data calculation.^26^

The study has some limitations. First, the AMASS requires users to validate the autogenerated reports as recommended for any analytical software.^26^ Second, the HIS data used in the study could not identify patients who were transferred from other healthcare facilities. Nonetheless, this allows our study to focus on comparing hospital-origin AMR BSI between participating hospitals while the transferred patients who had blood cultures positive for pathogens on admissions were classified as community-origin BSI in our study. Third, mortality (%) reported in this study was all-cause in-hospital mortality, and could be much lower than the true all-cause mortality because a preference to die at home is high in some regions in Thailand.^27^ The comparisons between mortality among patients with AMR BSI, antimicrobial-susceptible BSI and ‘no infection’^22–25^ are not within the scope of this study. Fourth, VRE was not included in the analysis because the proportion and frequency of VRE BSI in our study hospitals were still relatively low during the study period (https://doi.org/10.6084/m9.figshare.22339933). Fifth, comparing AMR data between hospitals will need to be performed carefully due to the difference in classes, types or levels of the hospitals, case-mix, blood culture utilization and other confounding factors.^28,29^ In addition, the total number of cases and deaths with AMR BSI in each hospital depends not only on AMS and IPC practices, but also on the size of the hospital (i.e. total number of hospital admissions and total number of patient-days at risk). Therefore, the frequency and mortality rate (e.g. per 100 000 admissions, per 100 000 patient-days at risk, and per 100 000 population per study period) should be calculated and used to compare the AMR burden between hospitals with similar levels of care and between areas. Sixth, formal qualitative and quantitative studies for feasibility of the AMASS and utility of the data were not performed. These will be conducted in the next phase of the AMASS implementation.

In conclusion, local and timely AMR data are crucial for local and national actions. Empowering local hospitals to analyse, validate and utilize their own AMR data in a timely manner and with minimal effort, by using analytical software such as the AMASS, is strongly encouraged.

## Supplementary Material

dlad088_Supplementary_Data

## Supplementary data

Figures S1 and S2 are available as Supplementary data at *JAC-AMR* Online.
