# Supplementary material for: Local and timely antimicrobial resistance data for local and national actions: the early implementation of an automated tool for data analysis at local hospital level in Thailand
Source: JAC Antimicrob Resist. 2023 Jul 15;5(4):dlad088. doi: 10.1093/jacamr/dlad088 (PMC10349292; doi:10.1093/jacamr/dlad088)
Supplement: dlad088_Supplementary_Data [file dlad088_supplementary_data.docx]

**Supplementary Figure 1. The proportion of AMR among patients with community-origin BSI in 15 tertiary-care hospitals (TCHs) and 10 secondary-care hospitals (SCHs) in Thailand in 2022**

**
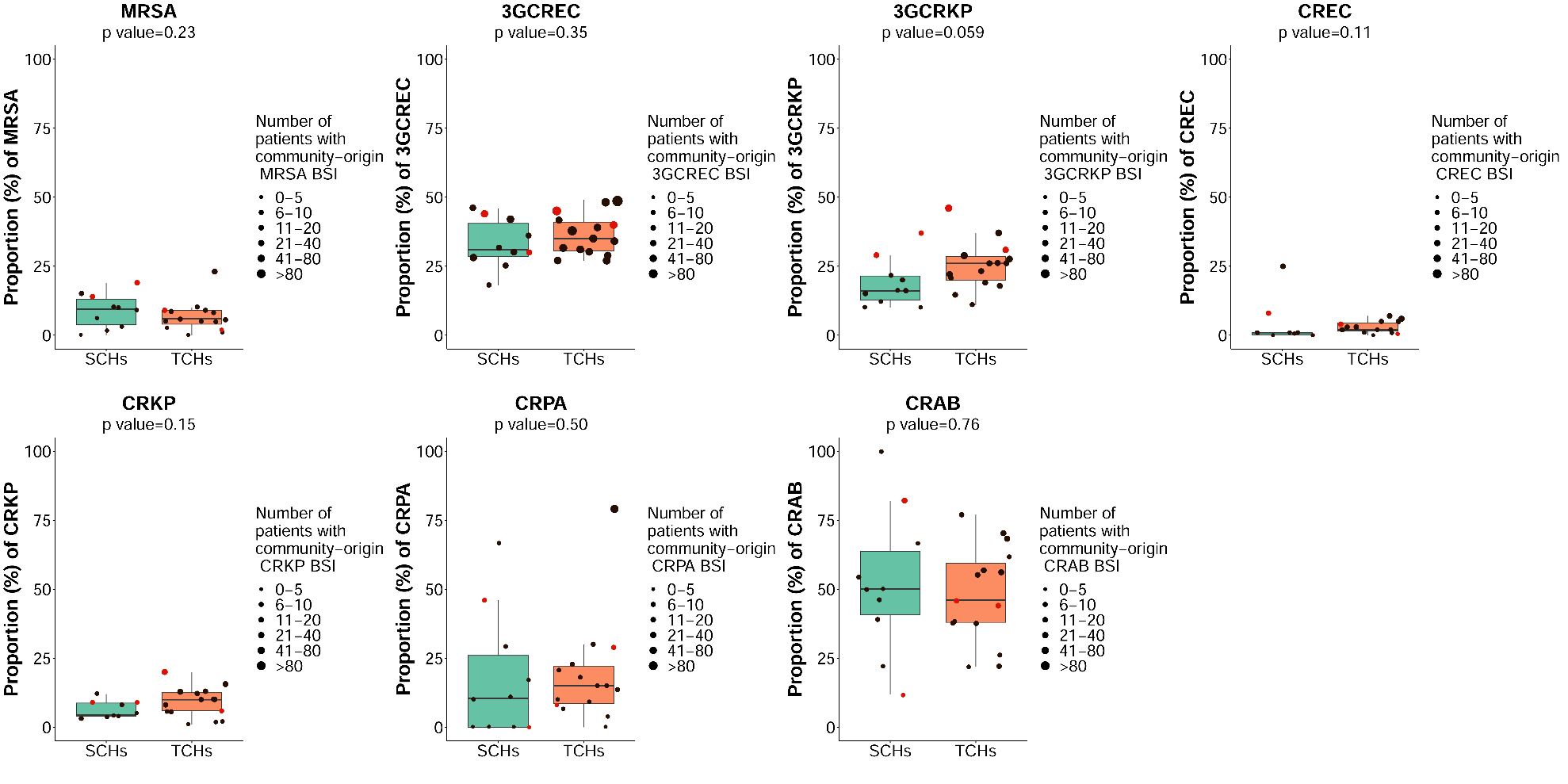
**

**Footnote of Supplementary Figure 1.** Each dot represents each hospital. Red dots represent anonymous hospital no. 22 and 24 (among TCHs) and anonymous hospital no. 16 and 25 (among SCHs)

**Supplementary Figure 2. The frequency of community-origin (CO) AMR BSI per 100,000 patients tested for CO BSI in 15 tertiary-care hospitals (TCHs) and 10 secondary-care hospitals (SCHs)**

**
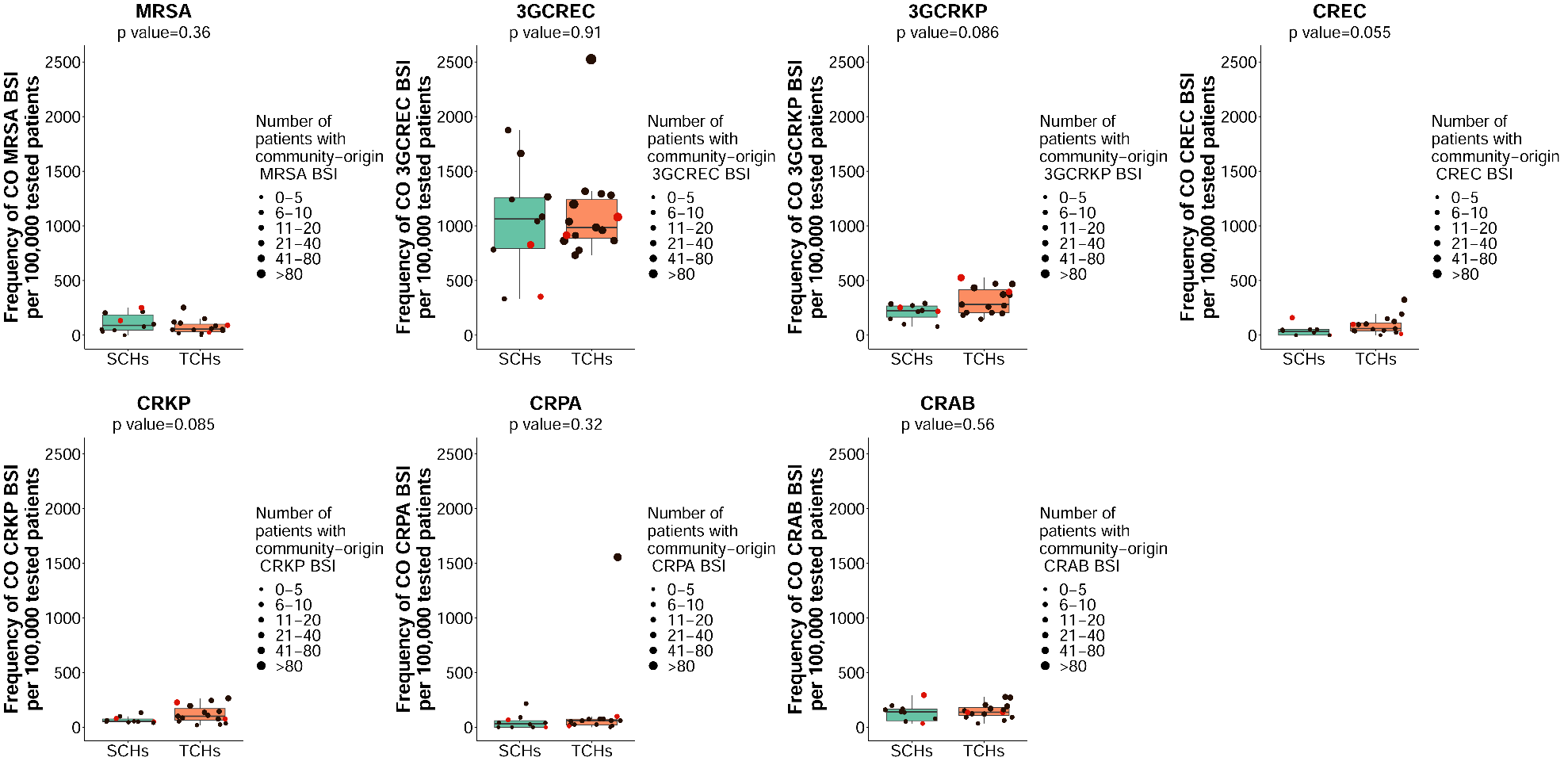
**

**Footnote of Supplementary Figure 2.** Red dots represent anonymous hospital no. 22 and 24 (among TCHs) and anonymous hospital no. 16 and 25 (among SCHs)
